# Dr. Pallas on the Management of Leeches

**Published:** 1826-07

**Authors:** 


16. *Leeches.* Dr. Pallas of the Military Hospital of Pampeluna, has made some experiments on the management of leeches, in order to render them serviceable for repeated applications.

When the rejection of the blood which these animals had drawn was procured by placing them on (wood?) ashes, a considerable majority were serviceable a second, and even a third time. But salt, ashes, tobacco, or other irritating substances which often seem to injure the leeches are by no means necessary to make them part with the blood which they may have taken. They will do this spontaneously, if they have the means of retiring and burying themselves in moist light earth. Dr. Pallas contrived a small tank, at the bottom of which was a perforated plate, allowing the escape of foul water but not of leeches. Within the tank an enclosure was formed with pieces of turf, and between this and the sides of the tank an interval of several inches was left all round. This was filled up with alternate layers of common mould, and clay. The tank was half filled with water, and several hundred leeches, which had already sucked, were placed in it. They put themselves into considerable agitation, became elongated, and discharged a portion of the blood, which they had taken, after which they made their way between the pieces of turf. A few sickly ones were the last to do so. In about a week eight died, but no living leeches remained in the water. Care was taken daily to water the earth, as well to keep it moist, as to wash out the blood which the leeches might have deposited in it. After fifteen days the tank was examined. The greater number of the leeches were found at the lower part, between the turf and the earth. They were motionless, contracted and short, resembling olives in form. They were surrounded by a thick coat of viscid matter, and the earth about them was soiled, shewing the necessity of frequent watering to prevent putrefaction, by which the leeches would be destroyed. Sixty of these leeches were taken out, washed, placed in clear water for a day, and then very efficiently employed in the service of the hospital.

